# Blood-Derived Biomarkers of Diagnosis, Prognosis and Therapy Response in Prostate Cancer Patients

**DOI:** 10.3390/jpm11040296

**Published:** 2021-04-13

**Authors:** Katalin Balázs, Lilla Antal, Géza Sáfrány, Katalin Lumniczky

**Affiliations:** Unit of Radiation Medicine, Department of Radiobiology and Radiohygiene, National Public Health Centre, 1221 Budapest, Hungary; balazs.katalin@osski.hu (K.B.); antal.lilla@osski.hu (L.A.); safrany.geza@osski.hu (G.S.)

**Keywords:** prostate cancer, radiotherapy, liquid biopsy, circulating tumour cells, extracellular vesicles, microRNAs, immune system, inflammation

## Abstract

Prostate cancer is among the most frequent cancers in men worldwide. Despite the fact that multiple therapeutic alternatives are available for its treatment, it is often discovered in an advanced stage as a metastatic disease. Prostate cancer screening is based on physical examination of prostate size and prostate-specific antigen (PSA) level in the blood as well as biopsy in suspect cases. However, these markers often fail to correctly identify the presence of cancer, or their positivity might lead to overdiagnosis and consequent overtreatment of an otherwise silent non-progressing disease. Moreover, these markers have very limited if any predictive value regarding therapy response or individual risk for therapy-related toxicities. Therefore, novel, optimally liquid biopsy-based (blood-derived) markers or marker panels are needed, which have better prognostic and predictive value than the ones currently used in the everyday routine. In this review the role of circulating tumour cells, extracellular vesicles and their microRNA content, as well as cellular and soluble immunological and inflammation- related blood markers for prostate cancer diagnosis, prognosis and prediction of therapy response is discussed. A special emphasis is placed on markers predicting response to radiotherapy and radiotherapy-related late side effects.

## 1. Introduction

Based on the 2020 cancer statistics of the International Agency for Research on Cancer (IARC) out of 19.3 million newly diagnosed cancers prostate cancer is ranked as the third most common among both sexes (constituting 7.1% of total cases). Regarding mortality rate it is on the eights place with approx. 375,000 deaths per year [1]. There are marked differences in the incidence of prostate cancer among various countries and races. It was reported that Japanese men living in Japan had very low prostate cancer incidence, while the incidence among USA resident Japanese increased and was at an intermediate level between Japanese living in Japan and European American men. Conversely, African American men have the highest incidence and mortality rates from prostate cancer within the United States [2,3]. These observations stress the importance of both genetic susceptibility and lifestyle in disease development and progression.

Among the main reasons for the increased prostate cancer mortality are the lack of reliable and effective prognostic biomarkers and methods which enable to recognise tumours in an early stage, to monitor individual therapy response more effectively, to sensitively detect minimal residual disease and development of distant metastasis as well as predict tumour relapse. These markers would allow patient stratification for optimal response rate to a certain therapy and enable the identification of those patients who are at increased risk for developing therapy-related side effects; thus, they are prerequisites for the development of efficient individualized anticancer treatment protocols.

At present diagnosis of prostate cancer is complex and is based on symptoms such as difficulties in urination, presence of blood in the urine or sperm, physical examination (including rectal digital examination), ultrasound examination, blood test to measure the prostate specific antigen (PSA) and tissue sample testing (biopsy). Due to the rather unspecific symptoms, early diagnosis of prostate cancer is not without problems and often the disease is only diagnosed in an advanced stage, where actually symptoms related to bone metastasis (bone pain and limb weakness caused by spinal marrow compression) are already present. While early detection of prostate cancer is fully curable, the efficiency of anti-cancer treatment in an advanced stage of the disease is very low.

Therefore, regular screening protocols in asymptomatic men with the purpose of identifying early-stage prostate cancer would be very important. Though, regular screening for prostate cancer has certain caveats. One such caveat is that more than 75% of PSA-positive tests (with blood PSA levels above 4 ng/mL, traditionally used as a cut-off value) are followed by a negative biopsy [4]. Biopsy can lead to infections [5], significant drop in quality of life [6] and can cause urinary, bowel and sexual dysfunctions persisting for several months [7,8]. Another caveat is the identification of indolent prostate cancer patients. Based on autopsy material, prostate cancers are identified at a much younger age (31–40 years) than clinically diagnosed in symptomatic patients. It appears that some prostate cancers may pass through a period of latency of up to 15 to 20 years, during which the disease is histologically present but it is completely asymptomatic. PSA-based screening of the population might result in over-diagnosed and therefore over-treated indolent prostate cancers resulting in serious side effects (such as incontinence and impotence), which would have caused no clinical consequences during a man’s lifetime if left untreated [9,10,11].

The issue of regular prostate cancer screening is a dilemma all over the world. Currently, in several European countries the main indications of annual prostate monitoring are age (men over 45 years) and/or family history. Nevertheless, the influence of family history for the risk of developing prostate cancer is recently being under revision due to studies with contradictory conclusions (Selkirk, Wang et al., 2015, Abdel-Rahman 2019). Increasing number of studies are investigating benefits, harms and cost-effectiveness of prostate cancer screening based on experimental and clinical data, clinical trials and model calculations. Two big studies are especially worth mentioning. One such study called the European Randomised Study of Screening for Prostate Cancer (ERSPC) randomized more than 180,000 men from Europe to analyse the longitudinal relationship between PSA values and biopsy (biopsy was carried out if blood PSA level was 3.0 ng/mL or higher) at regular intervals (every 4 years). After 13 years of follow-up the risk of prostate cancer mortality decreased 21% in the surveyed population compared to control group [12]. The other one called the Prostate, Lung, Colorectal and Ovarian (PLCO) carried out in the USA randomised more than 76,000 men using basically similar screening principles to the ERSPC study. Their final, updated conclusion, as a result of an extended follow up was that no significant difference was found in prostate cancer mortality in the screened group compared to control [13]. Thus, regarding the primary endpoint of the two studies, namely, to evaluate the predictive value of PSA screening in reducing prostate cancer mortality the two trials seem to reach contradictory conclusions. However, a recent re-analysis of both trials showed that the discordant results were due to differences in implementation and setting. After correcting them both studies reached the conclusion that screening could significantly reduce prostate cancer death [14]. These clinical trials highlight the overall positive balance of screening even using a marker (PSA) by far not optimal in detecting those patients which indeed should be treated for prostate cancer. More efficient, cost-effective and specific screening methodology is needed to reliably discriminate prostate cancer from benign alterations or other non-cancerous prostate diseases. Screening is especially important in races with an increased incidence of the disease (African American men) in order to reduce racial differences in prostate cancer survival [15].

Over the past decade, liquid biopsy investigations have received more and more attention. This minimal invasive method enables us to study a wide array of blood-based cellular and secreted soluble or vesicular markers, which offer a complex, comprehensive and real-time information on tumour stage, progression, tumour micro- and macro-environment, including the integrity of the anti-tumour immune response. These complex indicators might serve as prognostic and/or predictive markers able to predict patients’ outcomes, their response to particular therapies, forecast the formation of late therapy-related side effects (such as secondary tumours after radiotherapy) and ultimately to improve medical decision-making [16]. Blood-based markers of prognosis or therapy responsiveness are especially important in prostate cancer patients because of the high heterogeneity and molecular diversity of prostate cancer and because the prostate gland contains different subclones, which respond differently to treatments [17,18], so prostate gland biopsy can be misleading.

**Figure 1:**
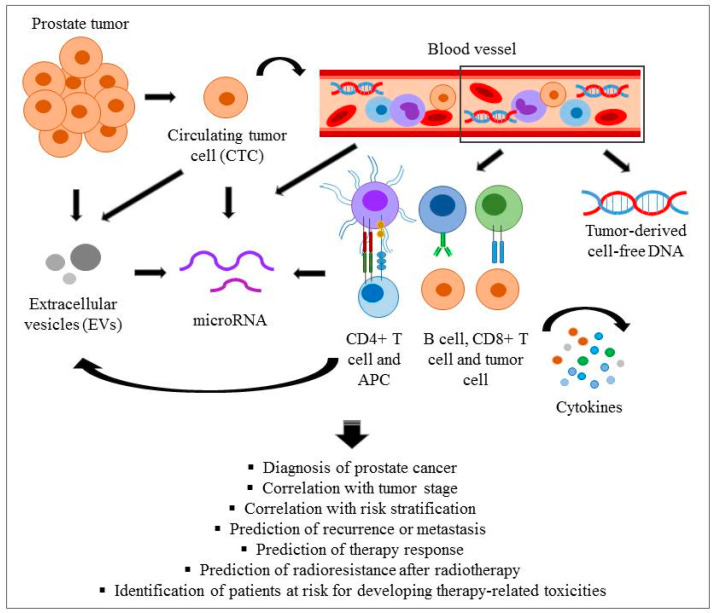
Overview of candidate blood-based liquid biopsy markers in prostate cancer patients.

In this review, we summarize the current knowledge on blood-based liquid biopsy analyses in prostate cancer focusing on disease- and therapy-related changes in PSA and related molecules, immune cells and immune- and inflammation-related secreted factors, circulating tumour cells (CTCs) and tumour-derived cell-free circulating nucleic acids as well as extracellular vesicles (EVs) and their micro-RNA (miRNA) content (Figure 1 and Table 1). We also discuss the relevance of these markers in radiotherapy-treated patients, in predicting their therapy-response or their risk for developing radiotherapy-induced late toxicities. Apart of blood-derived markers a large panel of urine and tissue-based potential biomarkers with prognostic and/or predictive value are identified. A few are already in the possession of clinical approval and some are still in an experimental stage. Though, in the present review we do not wish to focus on these. Detailed reviews such as [19,20,21,22,23,24] are available on these topics. For interested readers we advise consulting these publications.

This schematic picture summarizes the current knowledge on liquid biopsy analyses in prostate cancer focusing on circulating tumour cells (CTCs), immune cells and secreted factors such as tumour-derived cell-free circulating nucleic acids, cytokines and chemokines, as well as EVs released by prostate cancer cells, CTCs or various immune cells and their microRNA (miRNA) content.

## 2. Blood-Based Liquid Biopsy Marker Candidates

### 2.1. PSA and Related Molecules

At present, PSA is the most widespread and most accepted biomarker for prostate cancer monitoring; however, it lacks many of the qualities of an ideal tumour marker. PSA is a serine protease secreted physiologically by the prostate gland epithelium [25]. The total PSA (tPSA) is present in the serum in two forms, free (fPSA) and conjugated to serum proteins like alpha1 antichymotripsin, alpha2 macroglobulin and alpha1 protease inhibitor. Free PSA is comprised of pro PSA, benign PSA (BPSA) and intact PSA. Pro PSA is a zymogen precursor of PSA that comes in four different isoforms, as determined by the length of its pro leader peptide sequences [26]. Pro PSA is associated with cancer, BPSA with benign diseases whilst the association of intact PSA is currently unknown [27]. The free component represents about 5%–35% of tPSA [28]. The normal range of tPSA (commonly called as PSA) is 0–4 ng/mL in peripheral blood and levels above 10 ng/mL are considered pathological [29].

Monitoring regular variations in PSA level might serve as rough indicators of cancer progression (and regression after therapy) as well as disease recurrence. Since PSA is a tumour-associated antigen (TAA) and not a tumour-specific one, its specificity in prostate cancer diagnosis is not 100%, since inflammation, benign prostate hyperplasia (BPH) or other non-malignant disorders could also cause increased blood PSA levels, while normal PSA levels do not necessarily exclude the presence of a tumour. Furthermore, the age of cancer patients also influences the risk of cancer-specific death. With 7–10 ng/mL of PSA the risk of death is only 7% for men aged 50–59 but increases to 51% for men aged 80–89, 10 years after diagnosis [30].

As mentioned before, 75% of men with PSA levels above 4 ng/mL are not diagnosed with prostate cancer on biopsy. Only 26% of patients with PSA level within the “grey zone” of 4.1–9.9 ng/mL have cancer [31]. The PSA grey zone needs more accurate non-invasive diagnostic biomarkers to avoid the false-positive results because of benign changes. It was shown that PSA produced by prostate cancer cells escape degradation and occur in complexed form in the serum. When serum PSA is between 4 and 10 ng/mL and free to total PSA ratio (F/T ratio) is less than 25%, one should strongly suspect prostate malignancy, thus avoiding up to 20% of unnecessary prostate biopsies [32].

The so-called ‘prostate healthy index’ (PHI) reflects the ratio between total PSA, free PSA and pro PSA levels and prostate health index density (PHID) combines PHI parameters with prostate volume. These markers are clear improvement over PSA since they allow a better distinction between benign and malignant prostate gland hypertrophy and improve the prediction of high-grade and clinically aggressive prostatic tumours, especially in cases where PSA levels are in the grey zone [33,34,35,36,37,38].

The four kallikrein (4K) test measures tPSA, fPSA, intact PSA and human kallikrein-related peptidase 2 (hK2) in serum and is used to get a probability score for prostate cancer [39]. Similar to PHI, it improves the identification of overall and high-grade cancer and helps with reducing unnecessary biopsies [40,41]. Combined with PHI increases their diagnostic value [37]. Both the PHI and 4K test have been approved by the US Food and Drug Administration (FDA) to be used for prostate cancer screening. Recently two novel cancer-related glycoproteins, thrombospondin 1 (THBS1) and cathepsin D (CTSD) were proposed as blood-based biomarkers that outperformed PSA in distinguishing benign disease from prostate cancer in men with enlarged prostate gland [42]. A recently commercialized product—the Proclarix—incorporating THBS1, CTSD, tPSA and % of fPSA, combined with patient age, yielded a significantly better diagnostic accuracy compared to either PSA or % of fPSA alone in discriminating clinically significant from no or insignificant prostate cancer [43,44]. Though we have not found studies comparing the diagnostic efficiency of Proclarix with either PHI or 4K test.

### 2.2. Circulating Tumour Cells (CTCs) and Cell-Free Circulating Tumour DNA (cfDNA)

Prostate cancer metastasis is initiated by CTCs originating from the primary tumour transported through the blood or lymphatic system [45]. Some CTCs die in the circulation, others proliferate and form metastasis in distant organs [46]. Experimental models indicate that millions of tumour cells continuously circulate through the body, although only few of them can survive by evading the immune response and systemic therapies, reach a distant organ, proliferate and ultimately form metastases [47].

The number of CTCs in peripheral blood is very low, on average one CTC per one million peripheral blood mononuclear cells (PBMCs) [48], so their isolation is challenging. Several approaches have been reported for CTC detection, isolation and characterization in the peripheral blood of cancer patients such as two-stage microfluidic chip technology [49], acoustic separation of CTCs [50] and in situ hybridization (ISH) technology combined with immunomagnetic selection [51]. There are label-free techniques as well, which include size-based and density-based approaches [52] and methods based on Ficoll-Paque centrifugation [53], electrical property-based separation [54] and leukocyte depletion (anti-CD45 immunomagnetic negative selection) [55]. At present, the CellSearch™ method is an FDA-approved technology based on CTC characterisation used to predict the outcome of prostate cancer patients. It enriches CTCs from the peripheral blood using a magnetic ferrofluid containing antibodies against epithelial cell adhesion molecule (EpCAM), which is a common CTC marker. Cells are then stained for expression of cytokeratine (CK) 8, 18, and 19, all of which are intracellular structural proteins found in epithelial cells [56].

CTCs are critical for monitoring anti-cancer therapeutic efficacy such as drug screening [57], since resistance towards various chemotherapeutic agents remains a major clinical challenge [58]. Regular monitoring of CTCs can give a more complex and more realistic view of tumour heterogeneity than conventional biopsy [59]. Furthermore, CTCs are independent prognostic factors of progression-free survival (PFS) and overall survival (OS) in metastatic breast [60], colon [61] and prostate cancers [62] and their presence was implicated in worse cancer prognosis and outcome [63]. Patients with CTC numbers higher than 5 per 7.5 mL whole blood (as compared with the group with CTC numbers lower than 5 per 7.5 mL blood) had shorter median PFS and OS [64]. Quantification of CTCs has been proposed also as a potential surrogate endpoint to promote the selection of treatment algorithms [65] especially in advanced-stage prostate cancer disease, although the small number of cells detectable in the blood of prostate cancer patients and the lack of specific molecular determinants on CTCs indicative of therapy response have limited its clinical utility [66].

Not just CTC numbers can serve as prognostic markers but also the repertoire of their cell surface molecules which could also indicate the efficacy of various therapies such as radiotherapy or immune therapy. The EpCAM glycoprotein was initially described as one of the most commonly used protein CTC markers, however its level was shown to be downregulated during the dissemination of cancer cells from primary tumour [67]. Therefore, using CTCs as biomarkers of therapy response based purely on their immune phenotypical changes might be misleading because of their dynamic evolution during cancer progression [68].

Certain studies suggest that CTCs adopt different strategies to protect themselves from therapy-induced cell death, developing an epithelial to mesenchymal transition (EMT), grouping into clusters or switching between cancer stem cell state and differentiated cancer cell state. Not only individual CTCs, but also CTC clusters, their EMT and the presence of cancer-associated macrophage-like cells (CAMLs) in the blood are indicative for an increased risk of metastatic disease. Compared to single CTCs, CTC clusters may be more aggressive in forming distal metastasis [69]. CTC cluster size and number have been associated with lower overall survival in patients with breast, pancreatic, or prostate cancer [70,71,72]. These additional features of CTCs apart of their immune phenotypical changes have improved their prognostic value.

The programmed cell death protein (PD-1) and its ligand 1 (PD-L1) are major targets of the immune checkpoint inhibitor therapies in metastatic cancer diseases. PD-L1 expressing circulating epithelial tumour cells (CETCs) are described in 100% of prostate, 94.5% of breast, 95.4% of colorectal and 82% of lung cancer patients. Monitoring the frequency of PD-L1 positive CTCs could reflect individual patient’s response to anti-PD-1/PD-L1 therapy [59,73]. Expression of nuclear PD-L1 (nPD-L1) in the CTCs of prostate cancer patients was shown to significantly correlate with short survival rate [74]. In conclusion, the use of CTC-based models for risk assessment can improve standard cancer staging.

The effect of radiotherapy on CTCs is controversial. Martin et al. described that fractionated radiotherapy disrupted the tumour mass in non-small cell lung cancer (NSCLC), thus promoting the passage of tumour cells into the circulation [75]. In contrast, Budna-Tukan et al. analysed the number of CTCs in patients with non-metastatic high-risk prostate cancer with three different innovative CTC enumeration technologies before and after radiotherapy. They did not find any differences and significant therapy-related changes in CTC counts. These latter data do not support the hypothesis that radiotherapy leads to CTC release into the circulation in prostate cancer most probably because radiotherapy efficiently reduces tumour size in patients with prostate cancer, therefore the number of cancer cells in the circulation should also partially or totally decrease. It would be interesting to analyse this CTC number in patients with worse prognosis and metastatic disease as well. Furthermore, the reason for the difference between radiotherapy effects on CTCs in NSCLC and prostate cancer could be that these tumours respond differently to radiotherapy. Less effective radiotherapy leads to a suboptimal tumour response, and possibly an increase in CTC numbers [76].

Some patients undergoing prostate brachytherapy develop distant metastases despite the absence of local recurrence. Although micrometastases were not detected by radiographic images in these patients, cytokeratine positive or PSA positive cells were present in the bone marrow aspirates, which were considered disseminated tumour cells (DTCs) [77]. It has been proposed that in the early phases of radiotherapy, when cancer cells suffer only from sub-lethal damage, the quick increase of CTCs could, in principle, contribute to the development of distant metastases [76]. Another explanation is that surgical manipulation with needles being inserted into the prostate tissue during brachytherapy may pose a potential risk for haematogenous spillage of prostate cancer cells and play a role in distant metastases development [78].

Quantification of cell-free circulating tumour nucleotides in blood samples is another promising new molecular strategy for non-invasive tumour monitoring in prostate cancer [79]. CfDNA may be more suitable than CTCs to estimate therapy efficiency in patients with early-stage disease [80]. CfDNA originates from apoptotic or necrotic cells (including CTCs) or is actively secreted by cancer cells [81] and has been detected in human blood, urine and semen [82].

CTCs carry the complete mutation spectrum of the primary tumours and metastases, [16,83] and therefore genetic and transcriptional analysis of individual CTCs might enable personalized medical decisions for cancer therapy and provide insights into the biological processes involved in metastasis. Somatic mutations are detected in advanced or metastatic tumours, so they are unsuitable for monitoring primary, nonmetastatic disease [84,85]. Conversely chromosomal rearrangements represent an early stage of cancer pathogenesis [86]. The most common chromosomal rearrangement in prostate cancer present in approx. 50% of patients results from the fusion of the androgen-regulated gene, transmembrane protease serine 2 gene (*TMPRSS2*, chr21q22.2), with E-twenty-six (ETS)-related gene (*ERG*, chr21q22.3). This rearrangement is detectable in CTCs or as cfDNA and might be considered as a highly tumour-specific, non-invasive molecular biomarker for therapy assessment, risk stratification and relapse detection [79,87,88]. Furthermore, prostate cancer carrying this rearrangement can regulate the recruitment and infiltration of regulatory T cells (Tregs) in the tumour [89].

Gene expression analysis of CTCs of prostate cancer patients have revealed altered expression levels of eight metastasis-related metabolic genes, such as phosphoglycerate kinase 1 (*PGK1*) and glucose-6-phosphate dehydrogenase (*G6PD*) responsible for optimal glucose metabolism in CTCs. Their increased expression level in CTCs was associated with advanced tumour stage and metastasis proneness [90].

The clonal evolution of cancer cells can be traced at the level of CTC DNA or cfDNA by next generation sequencing or tumour mutation allele frequency analyses. The dynamics and relative abundance of the different clones are suitable to evaluate disease and metastasis heterogeneity, to monitor the emergence of resistance mechanisms as well as therapy resistance [91]. Quantification of cfDNA can reveal treatment ineffectiveness at an early stage of the treatment protocol and also avoid toxicity of ineffective overtreatment. Thus, patients can receive alternative therapies [92]. Since both CTCs and cfDNA can be retrieved basically in a non-invasive manner by blood collection, their regular follow up allows a more precise monitoring of disease progression and a better patient care as well.

### 2.3. Cellular and Soluble Immunological Markers

Systemic inflammatory conditions as well as the components of adaptive and innate immunity are involved in the initiation and progression of prostate cancer [93,94,95]. The role of chronic inflammation in this process involves multiple mechanisms such as: (a) mutagenesis caused by oxidative stress, (b) remodelling of the extracellular matrix, (c) recruitment of immune cells including tumour-associated neutrophils (TANs), tumour-infiltrating macrophages (TIMs), myeloid-derived suppressor cells (MDSCs), mast cells, as well as fibroblasts, and endothelial cells, (d) elevated secretion of cytokines and growth factors contributing to a proliferative and angiogenic environment [96,97,98].

Innate immune cells (macrophages, neutrophils) are triggered by foreign microbial and viral structures, known as pathogen-associated molecular patterns (PAMPs), or normal cellular constituents released upon injury and cell death, known as damage-associated molecular patterns (DAMPs), which are recognized by pattern-recognition receptors (PRRs), like the Toll-like receptor (TLR) family [99]. Innate immune cells are the main players in the early phase of the inflammation and affect tumour progression via intercellular signalling including cytokines and chemokines [100]. Tan et al. found in animal studies that the Cystein-X-Cystein (C-X-C) motif chemokine ligand 9 (CXCL9) regulated the host’s response to inflammation by recruiting leukocytes to the inflammatory environment and had important role in promoting prostate tumorigenesis [101]. Activation of the innate immune system implies upregulation of major histocompatibility (MHC) class I and II molecules on the surface of nucleated cells and antigen presenting cells (APCs) like macrophages, dendritic cells (DCs) and B lymphocytes and presentation of tumour associated antigens on their MHC molecules to naive T lymphocytes [102]. These processes induce the production of different inflammatory chemokines and cytokines, which in turn lead to the activation of both the cellular and humoral arm of the adaptive immune response [103].

An important step in prostate cancer development is tumour immune escape manifested among others in defects in antigen presentation (human leukocyte antigen (HLA) class I receptor deficiency on cytotoxic T cells), imbalance in T helper type 1 (Th1) and Th2 cytokine production leading to elevated levels of immunosuppressive cytokines such as interleukine-4 (IL-4), IL-6 and IL-10. Furthermore, induction of T cell death, T cell receptor dysfunction, prostate tumour infiltration with tolerogenic DCs and Tregs are also important indicators of an immune suppressing microenvironment and low tumour immunogenicity [104].

Granulocyte colony-stimulating factor (G-CSF), IL-1β, or tumour necrosis factor (TNF) secreted by tumour cells extend the lifespan of neutrophils and attract them to the tumour microenvironment, where they become immunosuppressive tumour-associated neutrophils, which stimulate proliferation of tumour cells and angiogenesis [105]. Macrophages are important in promoting growth and bone metastasis of prostate cancer [106]. Monocyte chemotactic protein (MCP)-1/C-C motif ligand (CCL)2 secreted by cancer cells recruits tumour-infiltrating macrophages and induces tumour progression [107]. MDSCs are the immature form of myeloid cells and they suppress anti-tumour immune responses in the tumour microenvironment. Tregs are among the most important immune cell populations suppressing antitumour immune responses [108]. Increased Treg infiltration in the tumour microenvironment through C-C chemokine receptor 4 (CCR4) [109] with the high expression level of the cytotoxic T-lymphocyte-associated protein 4 (CTLA-4) and PD-1 markers was linked with a poor prognosis in prostate cancer [110,111]. Several studies published that the number of Tregs showed significant correlation with the number of macrophages with tumour promoting M2 phenotype in the prostate cancer microenvironment and together they were associated with a worse clinical outcome [112,113]. Furthermore, isoforms of cluster of differentiation 44 (CD44) transmembrane glycoprotein receptor serve also as a poor prognostic factor in prostate cancer. This receptor has different isoforms. The standard isoform (CD44std) is expressed in normal epithelial cells [114], while the variant isoforms (CD44v) are highly expressed in several epithelial-type carcinomas [115]. Increased expression of CD44v (also known as CD44 variant 6) was associated with progressive disease and poor prognosis in prostate cancer [116].

Several immune and inflammatory genes harbouring single nucleotide polymorphisms (SNPs) associated with prostate cancer risk were identified, including pattern recognition receptors (macrophage scavenger receptor 1 or *MSR1*, *TLR1*, *TLR4*, *TLR5*, *TLR6*, and *TLR10*) [117,118,119,120]; antiviral genes (ribonuclease L or *RNASEL*) [121,122]; cytokines (macrophage inhibitory cytokine 1 or *MIC1*, *IL-8*, *TNF-α*, and IL-1 receptor antagonist or *IL1RN*) [123,124]; and the proinflammatory gene cyclooxygenase 2 (*COX-2*) [125].

Several of the identified gene expression signatures in prostate cancer are also immune-related, highlighting the importance of immune system in disease pathogenesis. Liong et al. developed a blood-based biomarker panel consisting of 7 mRNAs and demonstrated in 739 prostate cancer patients that the panel could identify men with aggressive prostate cancer. It is important to highlight that the majority of the differentially expressed genes were involved in immune processes [126]. Wallace et al. reported differential gene expression signatures in prostate cancer samples from African American and European American men and the majority of the differentially expressed genes was also immune related (immune response, defence response, antigen presentation, B/T cell function, cytokine signalling, chemokines, inflammatory response) [127]. The C-X-C motif chemokine receptor 4 (CXCR4) chemokine, previously linked to tumour metastasis [128] was differentially expressed between tumour and surrounding non-tumour tissue in African American men and the CXCR4 pathway was the highest-ranked pathway showing differential expression pattern among tumour and non-tumour tissue in African American men [127,129]. Clinical application of these gene expression signatures in therapy individualisation holds great promise. Though, we should mention that the so-far tested approaches for molecular characterisation of prostate cancer are based on biopsy tissues. In order to overcome serial biopsies, analysis of CTCs holds great promise in the non-invasive molecular profiling of prostate cancer and in determining both tumour- and patient-derived heterogeneity in disease progression and therapy response. A report on CTC-based liquid biopsy signatures with prognostic relevance and with implications in therapy decision has recently been published [130].

Radiotherapy can influence immune processes by increasing the expression of TAAs and DAMPs, as well as inducing cell death and secondary release of the proinflammatory cytokines and chemokines [131,132]. Radiotherapy-induced DNA, protein and lipid damage was shown to increase free radical levels in the circulation released by directly irradiated cells [133]. Gupta et al. found that IL-6, IL-8, TNF-α and transforming growth factor-beta (TGF-β) were the major mediators of ionizing radiation response in prostate cancer after radiation therapy influencing signalling pathways targeting transcription factors such as nuclear factor kappa B (NF-ĸB), activator protein-1 (AP-1) and signal transducers and activators of transcription (STATs). These transcription factors further enhanced expression of IL-1β and TNF-α [134]. Furthermore, radiotherapy also upregulated MHC class I on cancer cells, leading to the recognition of TAAs by cytotoxic T cells, enabling them to raise an antitumour response. Thus, prostate radiotherapy could potentially initiate a systemic, or ‘abscopal’ immune response, resulting in antitumorigenic responses in distant metastases [135]. According to Reits et al., the effect of γ-irradiation on MHC class I molecules could explain immune-mediated abscopal effects [136,137].

It was shown that, in patients responsive to androgen deprivation therapy (ADT), the baseline levels of certain immune markers such as IL-6, IL-10, granulocyte macrophage colony stimulating factor (GM-CSF) was significantly lower, and the level of certain pro-inflammatory cytokines (IL-5, interferon-γ (IFN-γ), TNF-α) during the course of the therapy was significantly higher than in patients resistant to ADT [138]. Both ADT and radiotherapy can damage the endothelium network in prostate cancer and vascular damage is part of radiotherapy-caused late toxicities [139]. It was reported that ADT downregulated vascular endothelial growth factor (VEGF) in normal tissue as well as in malignant prostatic tissue. In the absence of VEGF immature blood vessels underwent selective apoptosis and endothelial dysfunction [140]. Microvascular endothelial cell apoptosis after high dose irradiation constituted a primary lesion, developing into persistent endothelial dysfunction with microvessel collapse, endothelial cell activation and ultimately premature aging and senescence [139]. These effects impact normal tissue homeostasis leading to hypoxia and consequent ischemia, as well as inflammation and fibrosis. The early phases of fibrogenesis after irradiation were characterized by the upregulation of pro-inflammatory cytokines such as TNF-α, IL-1 and IL-6 and many growth factors in the irradiated tissue [141]. It was shown that a complex balance between TGF-β [142] and its downstream effector connective tissue growth factor (CTGF) [143], the antifibrotic proteins such as TNF-α and IFN-γ was important in this process [144].

Prostate cancer is among the more radioresistant malignant tumours [145]; the disease recurs in 30%–40% of prostate cancer patients receiving radiation therapy [146]. A retrospective study investigated the overexpression of 24 genes (DNA damage regulated autophagy modulator 1 or *DRAM1*, keratin 14 or *KRT14*, protein tyrosine phosphatase, non-receptor type 22 or *PTPN22*, zinc finger matrin-type 3 or *ZMAT3*, Rho GTPase activating protein 15 or *ARHGAP1*, *IL-1B*, anillin actin binding protein or *ANLN*, ribosomal protein S27a or *RPS27A*, melanoma associated antigen mutated 1 or *MUM1*, topoisomerase (DNA) II alpha or *TOP2A*, cyclin dependent kinase inhibitor 3 or *CDKN3*, G protein subunit gamma 11 or *GNG11*, haematopoietic cell-specific Lyn substrate 1 or *HCLS1*, denticleless E3 ubiquitin protein ligase homologue or *DTL*, IL-7 receptor or *IL-7R*, ubiquitin like modifier activating enzyme 7 or *UBA7*, NIMA related kinase 1 or *NEK1*, CDKN2A interacting protein or *CDKN2AIP*, apurinic/apyrimidinic endonuclease 2 or *APEX2*, kinesin family member 23 or *KIF23*, sulfatase 2 or *SULF2*, polo like kinase 2 or *PLK2*, essential meiotic structure-specific endonuclease 1 or *EME1*, and bridging integrator 2 or *BIN2*) related to radiotherapy and DNA damage-response and found that their expression signatures predicted response to radiotherapy and radioresistance in prostate cancer patients and helped specifically with selecting patients profiting from radiotherapy [147]. Further predictive markers of radioresistance are oxidative stress markers such as lipid peroxidation 4-hydroxylnonenal (4HNE) or 3-nitrotyrosine (3NT) [148,149]. Preclinical studies showed that hypoxia lead to a radioresistant and metastatic phenotype of prostate tumours [150]. Extracellular vesicles could mediate hypoxia-induced prostate cancer progression, enhanced the invasiveness and stemness of prostate cancer cells and increased the level of signalling molecules such as TGF-β2, TNF-lα, IL-6, tumour susceptibility gene 101 (TSG101), protein kinase B (PKB or Akt), integrin-linked kinase 1 (ILK1), matrix metalloproteinase (MMP), and β-catenin [151]. High level of the IL-8 (CXCL8) chemokine was also associated with increased radioresistance [152]. Sequence variants of several genes such as ataxia-teleangiectasia mutated (*ATM)*, breast cancer type 1 susceptibility protein (*BRCA1)*, H2A histone family member X (*H2AFX)* and mediator of DNA damage checkpoint protein 1 (*MDC1)* were linked to increased radiosensitivity and could distinguish prostate cancer patients with high radiation toxicity from those with low toxicity [153,154]. Langsenlehner et al. investigated 603 patients treated with three-dimensional conformal radiotherapy and found that single nucleotide polymorphisms in the X-ray repair cross-complementing protein 1 (*XRCC1*) gene was associated with radiation-induced late toxicity in prostate cancer patients [155].

### 2.4. Extracellular Vesicles

Extracellular vesicles (EVs) received considerable attention in recent years because of their role in intercellular communication. EVs are phospholipid bilayer membrane-coated vesicles released by most cell types in physiological and pathological conditions [156]. Since EVs are highly heterogeneous in size, biogenesis, function, content, membrane markers, and so forth [157], we use “extracellular vesicle” as a generic term to describe any type of membrane-coated vesicles (e.g., exosomes, microvesicles, microparticles, apoptotic bodies, etc.) released into the extracellular matrix. A common feature of all types of EVs is their complex cargo consisting of various bioactive molecules, like proteins, lipids, DNA-fragments, different species of RNAs. These molecules are protected by the lipid membrane of the EVs and thus they are transported in an intact and biologically functional form between cells [156,158,159].

The release of EVs from cells and their journey throughout the body is not random, several regulated mechanisms underlie this process. It was shown that cancer cells produced more EVs compared to normal cells [160]. This might be partly due to the acidic environment characteristic for many cancers. Several studies demonstrated that the extracellular pH was an important modifier of EV traffic, since low pH altered EV membrane fluidity [161] and increased EV release and uptake [162]. Under chronic hypoxia prostate cancer cells secreted more EVs as a survival mechanism to remove metabolic waste [163]. Tumour-derived EVs played a key role in tumour cell growth and in the crosstalk between cancer cells and the tumour microenvironment (TME), contributing to the development of a cancer-supportive microenvironment, angiogenesis [164,165] and metastasis [166,167].

Since tumour derived EVs have specific cargos, which differentiate them from EVs released under physiological conditions, and given the fact that they are released into various human body fluids (e.g., blood, saliva, urine, amniotic fluids, sperm, bile, etc.) [158], EVs represent a source of biomarkers for the early detection of cancer, therapeutic planning and monitoring. EVs can transmit their information to recipient cells in several ways, such as (a) transfer of bioactive molecules which regulate signalling pathways in recipient cells; (b) receptor shuttling to alter cellular activities; (c) delivery of fully functional proteins to accomplish specific functions in target cells; (d) and providing new genetic information with various type of nucleic acids to gain new traits [168]. Accordingly, different types of biomolecules within the EV cargo can serve as prognostic and predictive biomarkers in cancer. Increased plasma EV levels in prostate cancer patients were reported by several studies [169,170,171,172] but reports are contradictory whether prostate cancer cell-derived EVs could be distinguished from total plasma EVs based on the presence of prostate-specific membrane antigen (PSMA) on the EV membrane. Plasmatic EVs expressing both CD81 and PSA were significantly higher in prostate cancer patients compared to either healthy controls or patients with BPH, reaching 100% specificity and sensitivity in distinguishing prostate cancer patients from healthy individuals [172]. Biggs et al. reported that prostate-specific plasma EV (identified based on PSMA expression on the EV membrane) numbers were suitable to identify prostate cancer patients with high risk, and those with metastatic disease [169]. On the other hand, Joncas et al. found that PSMA expression on plasma EVs was not a reliable marker for the identification of prostate cancer cell-specific EVs. Their conclusion was based on the proteomic analysis of PSMA-enriched EVs, in which no cancer-specific proteins could be identified [170].

Investigation of plasma EV cargo, mainly their protein and RNA content is receiving much attention as diagnostic and prognostic tools in prostate cancer. EVs isolated from either plasma or urine could be utilized to monitor prostate cancer stages, to discriminate high-grade from low-grade prostate cancer and benign disease, thereby reducing the number of unnecessary biopsies. Although it is not a blood-based marker, it is important to mention that ExoDx Prostate, a commercialized, urine-based test evaluates 3 EV-derived mRNAs, used to identify high-grade prostate cancer in patients with previous negative biopsies or with low initial PSA values [173].

Survivin is an apoptosis inhibitor selectively expressed in different tumours, including prostate cancer, and its main role is to promote cancer cell survival and protect cancer cells from apoptosis. It was shown that survivin was present in EVs secreted by prostate cancer cells and survivin levels in plasma-derived EVs from newly diagnosed prostate cancer patients (both early-stage and advanced cancers) and patients who relapsed after chemotherapy were significantly increased. These findings indicate that plasma EV-derived suvivin might be a promising liquid biopsy marker for the early diagnosis and systemic monitoring of prostate cancer [174].

Lundholm et al. found that NKG2D ligand-expressing prostate tumour-derived EVs selectively induced the downregulation of NKG2D on natural killer (NK) and CD8+ T cells, leading to damaged cytotoxic T cell function in vitro. Consistently with these data, surface NKG2D expression on circulating NK and CD8+ T cells was significantly decreased in patients with castration-resistant prostate cancer (CRPC) compared to healthy individuals [175]. These findings suggest that prostate tumour-derived EVs promote immune suppression and tumour escape by acting as down-regulators of the NKG2D-mediated cytotoxic response in prostate cancer patients.

Androgen receptor splice variant 7 (AR-V7) was associated with resistance to hormonal therapy in castration-resistant prostate cancer and plasma-derived EVs were shown to contain AR-V7 RNA [176]. Validation of AR-V7 as a potential target for treatment of CRPC could make it a clinically predictive biomarker of resistance to hormonal therapy and facilitate the decision-making process and therapy planning in these patients. Additionally, another study suggested that EV AR-V7 RNA was correlated with lower level of sexual steroid hormones in CRPC patients with a poor prognosis [170].

### 2.5. MicroRNAs

RNA content of EVs is considerably different from their parent cells, suggesting that cells can selectively sort their species of RNA into EVs, including small non-coding RNAs such as miRNAs or miRs with important regulatory functions on protein expression. Each miRNA regulates multiple target messenger RNAs (mRNAs). They control protein expression through the degradation of mRNAs or the inhibition of protein translation of target mRNAs by binding to the 3′-untranslated region (UTR). In view of their complex regulatory ability, it is not surprising that abnormal miRNA expression has been described in the pathogenesis of several diseases including cancer. Incorporation of miRNAs into EVs or binding to RNA-binding protein complexes increases their stability and protects them from degradation by various environmental factors [177]. Therefore, miRNAs are very stable in serum, plasma and other biofluids and are resistant to boiling, pH change, repeated freeze-thaw cycles, and fragmentation by chemical or enzymes [178], making them ideal biomarker candidates for the diagnosis, prognosis, and therapeutic planning in cancer disease, including prostate cancer. So far miRNA research in the prostate cancer field mainly focused on the characterization of differentially expressed miRNAs or miRNA panels involved in tumour progression [179]. Relatively few clinical trials have been conducted to date to explore miRNAs as indicators of prognosis or prediction of therapy response (Table 1).

Recently, plasma-derived EVs have proved to be better sources for miRNAs than unfractionated plasma/serum for certain but not all miRNAs. EV-incorporated miR-200c-3p and miR-21-5p could differentiate between prostate cancer and BPH, similarly EV-incorporated Let-7a-5p level could distinguish prostate cancer patients with Gleason score above 8 from those with Gleason score below 6. Both EV-incorporated and free miR-375 in the blood is an important miRNA biomarker candidate in prostate cancer. Huang et al. found that plasma EV-derived miR-375 and miR-1290 could predict overall survival for CRPC patients [180]. Expression of miR-141 and miR-375 increased in the blood of high risk or metastatic CRPC patients [181,182]. Other groups also confirmed miR-375 as important diagnostic marker but only if tested in the whole plasma [183]. Blood miRNAs were shown to discriminate between prostate cancer and BPH, though studies differ on the type and source of miRNAs. In one study overexpression of plasma-derived EV-containing miR-10a-5p and miR-29b-3p, while in another one downregulation of plasma-derived free hsa-miR-221-5p and hsa-miR-708-3p were indicative of prostate cancer but not BPH [184,185]. Correlated expression levels of miR-20a, miR-21, miR-145, and miR-221 [186], miR-17, miR-20a, miR-20b, miR-106a [187] as well as miR-16, miR-148a and miR-195 [188] in the plasma could significantly distinguish high risk patients from those with low risk and some of these miRNAs were shown to confer an aggressive phenotype upon overexpression in vitro as well as an accelerated biochemical recurrence [187]. Fredsoe et al. validated a blood-based miRNA diagnostic model comprising of 4 miRNAs (miR-375, miR-33a-5p, miR-16-5p and miR-409-3p), called bCaP, in 753 patients with benign prostate lesions and multiple stages of prostate cancer and showed that combined with PSA, digital rectal examination and age bCaP predicted the outcomes of biopsies better than PSA alone [189].

An important miRNA cluster with predictive value towards therapy response is formed by miR-205 and miR-31. These miRNAs regulate apoptosis in prostate cancer cells by targeting antiapoptotic proteins Bcl-w and E2F6 and they are downregulated in prostate cancer cell lines derived from advanced metastatic cancers. It was shown that their decreased expression could contribute to resistance to chemotherapy-induced apoptosis making them key targets to improve prostate cancer response to chemotherapy [190]. In this context, upregulated plasma miR-205 expression in metastatic CRPC was associated with a lower Gleason score and a lower probability of both biochemical recurrence and clinically evident metastatic events after prostatectomy [181].

Several studies highlight the prognostic and predictive value of circulating miRNAs secreted by other than prostate cancer cells, most probably reflecting a systemic response. Bone marrow mesenchymal stem cells EV-derived miR-205 contributed to repress prostate cancer cell proliferation, invasion, migration and enhance apoptosis, which suggests that miR-205 could be a valid prognostic marker and a potential therapeutic target in prostate cancer [191]. It was shown that tumour-associated macrophages (TAM)-derived EVs with increased miR-95 content could mediate prostate cancer progression by promoting proliferation, invasion, and EMT [192].

Ionizing radiation is an important exogenous factor, which modifies miRNA expression in cells, including cancer cells. Altered miRNA expression patterns can influence cancer cell radioresistance and consequently lead to changes in radiation response. There are relatively few studies, which investigate miRNA expression profile changes induced by irradiation in prostate cancer patients, most studies are mainly in vitro investigations. A comprehensive review of miRNA expression alterations after various irradiation schedules in different prostate cancer cell lines was prepared by Labbé et al. The authors also summarize the most important radiotherapy-regulated cellular mechanisms in which miRNAs are involved [193].

MiR-106a and miR-20a overexpression conferred radioresistance to prostate cancer models by increasing clonogenic survival after radiotherapy [187,194]. In another study using prostate cancer cell lines with different intrinsic radiosensitivity miR-200, miR-221, miR-31 and miR-4284 were found to correlate with clonogenic survival of cell lines after irradiation [195]. Increased miR-21 and miR-146a/155 levels were found in radiotherapy-treated prostate cancer patients with acute genitourinary side effects, indicating their potential to predict radiotherapy-related toxicities [196].

Gong et al. showed that circulating miR-145 levels were increased in prostate cancer patients responsive to neoadjuvant radiotherapy indicating that miR-145 might serve both as a predictive marker of therapy response and a novel therapeutic agent able to enhance the efficacy of radiotherapy [197]. MiR-93 and miR-221 plasma levels decreased significantly after either radical prostatectomy or radiotherapy but did not change after ADT and miR-93 significantly correlated with Gleason score in a cohort of 68 prostate cancer patients compared to the observational cohort (*n* = 81) [198]. Two studies investigated EV-miRNAs as markers of therapy efficacy. The study by Li et al. identified a panel of 9 serum-derived EV-miRNAs, which could predict therapeutic benefit of carbon ion radiotherapy based on their baseline values. Post-therapy levels of miR-654-3p and miR-379-5p were associated with therapy efficacy [199]. Another study identified hsa-let-7a-5p and hsa-miR-21-5p as increased only in high-risk prostate cancer patients after radiotherapy compared to intermediate-risk patients [200]. It is important to highlight that candidate miRNAs in the two studies did not overlap, which might be due to different patient enrolment criteria, treatment protocol and sampling time after therapy. A further limitation of the cited studies is the low number of enrolled patients (*n* = 8 and 11, respectively), thus data must be confirmed on larger patient cohorts as well.

**Table 1:** Summary of representative human studies investigating blood-derived liquid biopsy markers as biomarkers in prostate cancer patients.

Clinically Approved or Commercialized Biomarkers				
Biomarker Types	Biological Sample	Indicative for	Patient Numbers and Characteristics	References Or Clinical Trials.gov ID
PHI (total/free/pro PSA)	Plasma	- discrimination of prostate cancer and BPH patients; - prediction of high-grade and clinically aggressive prostatic tumours	892 men with no history of prostate cancer, normal rectal examination, prostate specific antigen between 2 and 10 ng/mL	[34] (Approved by FDA)
4K (Four kallikrein) test	Blood (serum)	- discrimination of prostate cancer and BPH patients	392 prostate cancer patients with PSA ≥ 3.0 ng/mL	[39] (Approved by FDA)
Proclarix (THBS1, CTSD)	Blood	- aid in the decision-making process before biopsy	955 prostate cancer patients	[43] (Commercialised)
CellSearch^TM^ CTC isolation	Blood	- prognostic factors of PFS and OS in metastatic prostate cancer	6081 patients with CRPC	[201] (Approved by FDA)
**Biomarkers in clinical trial**				
MDSCs	Blood	- therapy response indicator	300 patients, age ≥ 18, histological diagnosis of prostate cancer	NCT03408964 (Recruiting)
Antioxidant enzymes, oxidative stress markers, DNA damage in leukocytes	Blood	- patients at high risk for developing prostate cancer	40 patients with PSA ≥ 4.0 ng/mL; fPSA < 18%; PSA velocity > 0.75 ng/mL within the past year	NCT00898274 (Completed)
NK cells	Blood	- correlation between the level of NKp30 and NKp46 receptor-activators expression on the surface of NK cells	30 patients with metastatic prostate cancer; age ≥ 18	NCT02963155 (Active, not recruiting)
CTCs	Blood, plasma, PBMCs	- early markers of prostate cancer relapse and early metastases detected by PSMA-positron emission tomography (PET), who need further assistance in treatment decisions	50 patients in good general health and an expected life expectancy of >10 years diagnosed with prostate cancer relapse and positive lymph nodes as seen on PSMA-PET;	NCT04324983 (Recruiting)
Androgen receptor (*AR*), Phosphatase, tensin homolog (*PTEN*), *AR-V7* and other gene expression biomarkers in CTCs	Blood, Formalin-fixed paraffin-embedded (FFPE) sample	- predictive of outcome of activity of cabazitaxel treatment in CRPC	94 patients with metastatic CRPC; age ≥ 18	NCT03381326 (Active, not recruiting)
Tissue damage, CTCs	Blood, plasma	- prostate cancer patients at highest risk of radiotherapy-related complications	68 patients with prostate adenocarcinoma; age ≥ 18	NCT02941029 (Completed)
CTCs	Blood	- decrease the number of unnecessary prostate biopsies	500 patients, age ≥ 18; subjects with a PSA 4.00–10.99 ng/mL receiving biopsy within 3 months	NCT03488706 (Recruiting)
Immune checkpoint biomarkers (PD-L1, PD-L2, B7-H3, and CTLA-4) on CTCs	Blood	- metastatic prostate cancer	38 patients with histologically confirmed prostate adenocarcinoma; age ≥ 18 years;	NCT02456571 (Completed)
CTCs, cfDNA	Blood, plasma, tissue	- markers of drug resistance	24 patients with histologically confirmed prostate adenocarcinoma; increase PSA value over a baseline measurement	NCT02370355 (Terminated—Sponsor decided not to pursue study)
CTCs, cfDNA, exosomes	Blood	- discrimination of prostate cancer and non-cancer controls, identification of high-risk patients	320 men over 40 suspicious of prostate cancer; with PSA ≥ 4 and designated for biopsy	NCT04556916 (Recruiting)
Gamma H2AX Positivity	Blood	- prostate cancer cells response to radiotherapy	10 patients, age ≥ 18; histologically confirmed prostate adenocarcinoma	NCT02981797 (Completed)
TNF-α, IL-1β, IL-2, IL-2 CD25 Soluble Receptor, IFN-γ, IL-4, IL-5, IL-6, IL-8, IL-10, IL-12, IL-13	Blood, urine	- different types of cancer treatment can elicit different systemic immune responses from the body’s immune system	40 patients with histologically confirmed diagnosis of adenocarcinoma of the prostate	NCT03331367 (Completed)
170 clinically relevant SNPs	Saliva, blood, urine	- incidence and aggressiveness of prostate cancer	4700 patients, aged 55 to 69; caucasian ethnicity; WHO performance status 0–2	NCT03857477 (Recruiting)
PSA and 40 SNPs	Blood	- risk assessment and early detection of prostate cancer	5000 patients	NCT01739062 (Active, not recruiting)
DNA-repair gene defects	Saliva, Blood, Archival Tumor Tissue	- DNA-repair gene defect status of patients with metastatic prostate cancer	10,000 patients with histologically confirmed prostate adenocarcinoma	NCT03871816 (Recruiting)
miRNA expression of prostate cell-derived exosomes	Blood	- disease progression and relapse	600 patients with elevated PSA or patients with diagnosed prostate cancer; age ≥ 18;	NCT03694483 (Recruiting)
miRNA panel	Blood	- predictive value of miRNA and ARV7 status in treatment efficacy	46 CRPC patients with biochemical or clinical progression under hormone therapy; age ≥ 18;	NCT04188275 (Recruiting)
five prevalent exosomal miRNAs	Blood	- predicting duration of response to ADT	60 patients with histologically confirmed prostate adenocarcinoma; testosterone level > 30ng/mL; age ≥ 18;	NCT02366494 (Active, not recruiting)
miRNA	Not Provided	- predicting prostate cancer outcome	300 patients with clinically localised high risk prostate cancer scheduled for radical prostatectomy	NCT01220427 (Terminated)
**Biomarkers in experimental phase**				
PD-L1 expressing CTCs/CETCs	Blood	- indicates the efficacy of anti-PD-1/PD-L1 therapy	27 patients	[59]
Nuclear PD-L1 (nPD-L1) in CTCs	Blood	- prognostic indicator (short survival rate)	30 metastatic prostate cancer patients	[74]
CTLA-4 on Tregs	PBMCs	- immune suppression	32 patients	[202]
IL-4, IL-6, IL-10	Serum	- indicator of hormone refractory prostate cancer	18 hormone sensitive prostate cancer patients	[203]
SAMSN1, CRTAM, CXCR3, FCRL3, KIAA1143, KLF12, TMEM204	Blood mRNA	- indicator of aggressive prostate cancer	739 patients	[126]
SNPs of *TLR1*, *TLR4*, *TLR5*, *TLR6*, *TLR10*	Blood	- prostate cancer risk	18,018 US men (from the ongoing Health Professionals Followup Study)	[117]
SNPs of *MSR1*	Blood	- prostate cancer risk	83 Swedish prostate cancer patients	[118]
SNPs of antiviral genes (*RNASEL*)	Blood	- prostate cancer risk	101 prostate cancer patients with a family history of prostate cancer	[121,122]
SNPs of cytokines (*MIC1*, *IL-8*, *TNF-α*, and *IL1RN*	Blood	- prostate cancer risk	1383 prostate cancer patients, 779 controls	[123,124]
SNP of *COX-2*	Blood	- prostate cancer risk	506 prostate cancer and 506 controls	[125]
*ATM*, *BRCA1*, genes	Peripheral blood lymphocytes	- discrimination of patients with high and low radiation toxicity	37 prostate cancer patients	[153]
SNP of *XRCC1*	Blood	- radiation-induced late toxicity	603 prostate cancer patients	[155]
miR-141, miR-375	Serum	- high risk; - high Gleason score; - lymph node positivity	7 metastatic, 14 localized prostate cancer + 2 validation studies in different prostate cancer risk groups (*n*_1_ = 45 and *n*_2_ = 71)	[182]
miR-24, miR-26b, miR-30c, miR-93, miR-106a, miR-223, miR-451, miR-874, miR-1207, miR-5p, miR-1274a	Serum	- diagnosis of prostate cancer; correlation with prognosis	36 prostate cancer, 12 healthy controls	[204]
miR-26a, miR-32, miR-195, miR-let7i	Serum	- discrimination of prostate cancer and BPH patients; - correlation with Gleason score; surgical margin positivity	37 localized, 8 metastatic prostate cancer, 18 BPH, 20 healthy controls	[205]
miR-375, miR-141, miR-378, miR-409-3p	Serum	- correlation with diseases status	26 metastatic CRPC, 28 localized low-risk, 30 high-risk prostate cancer	[206]
miR-141, miR-298, miR-346, miR-375	Serum	- diagnosis of prostate cancer; - prediction of biochemical relapse	25 metastatic CRPC, 25 healthy controls	[207]
miR-16, miR-148a, miR-195	Plasma	- high risk; - high Gleason score; high PSA level;	79 prostate cancer patients, 33 healthy controls	[188]
miR-16, miR-21, miR-126, miR-141, miR-151-3p, miR-152, miR-200c, miR-205, miR-375, miR-423-3p	Plasma	- Gleason score; - lymph node involvement; time to tumour recurrence; - PSA level; - probability of biochemical recurrence in 5 years; - metastasis;	25 metastatic CRPC and 25 localized prostate cancer	[181]
miR-20a, miR-21, miR-145, miR-221	Plasma	- high risk versus intermediate or low risk prostate cancer patients	52 Low risk, 21 intermediate risk, 9 high risk prostate cancer patients	[186]
let-7c, let-7e, miR-30c, miR-622, miR-1285	Plasma	- diagnosis of prostate cancer; - discrimination from BPH	tested on 25 prostate cancer, 12 BPH, validated on 80 prostate cancer, 44 BPH, 54 healthy control	[208]
miR-375, miR-33a-5p, miR-16-5p, miR-409-3p	Plasma	- discrimination between benign prostate lesions and multiple stages of prostate cancer	753 patients (144 BPH, 464 prostate cancer for training + 145 for test)	[189]
miR-17 miR-20a miR-20b miR-106a	Plasma	- discrimination of high risk versus low risk prostate cancer patients	44 high risk, 31 low risk prostate cancer patients	[187]
miR-93, miR-221	Plasma	- prediction of therapy efficacy (radiotherapy, radical prostatectomy)	149 patients (68—treated, interventional cohort, 81—observational cohort)	[198]
let-7a, miR-141, miR-145, miR-155	Whole blood	- discrimination of prostate cancer versus BPH	75 prostate cancer, 27 BPH	[209]
hsa-miR-221-5p, hsa-miR-708-3p	Whole blood	- discrimination of prostate cancer versus BPH	115 prostate cancer, 39 BHP	[185]
miR-493-5p, miR-323a-3p, miR-411-5p, miR-494-3p, miR-379-5p, miR-654-3p, miR-409-3p, miR-543, miR-200c-3p	Serum EV	- prediction of therapeutic benefit of carbon ion radiotherapy; prediction of therapeutic efficacy	8 patients, localized cancer	[199]
hsa-let-7a-5p, hsa-miR-21-5p	Serum EV	- prediction of radiotherapy efficacy	11 patients (6 high-risk, 5 intermediate risk)	[200]
miR-10a-5p miR-29b-3p miR-99b-5p	Plasma EVs	- discrimination of prostate cancer versus BPH	18 prostate cancer, 7 BPH	[184]
miR-375, miR-1246, miR-1290	Plasma EVs	- correlation with overall survival;	screening in 23 CRPC, validating in 100 CRPC	[180]
Let7a-5p, miR-21-5p, miR-200c-3p, miR-375	Plasma, EVs	- discrimination of prostate cancer from BPH	50 prostate cancer, 22 BPH	[183]
miR-107, miR-130b, miR-141, miR-181a-2, miR-301a, miR-326, miR-331-3p, miR-432, miR-484, miR-574-3p, miR-625, miR-2110	Plasma-derived EVs, serum-derived EVs, urine	- diagnosis and staging of prostate cancer	78 prostate cancer, 28 healthy controls	[210]
miR-21 miR-146a miR-155	Blood PBMCs	- prediction of radiotherapy-related toxicities	15 prostate cancer, 9 with and 6 without acute gastro-urinary toxicity	[196]

## 3. Conclusions

Table 1 summarizes the most important liquid biopsy-based biomarkers or biomarker candidates either already approved for clinical use or investigated in ongoing clinical trials or still in experimental phase. The high number of studies focusing on different cellular and secreted blood components as candidate liquid biopsy markers demonstrates the need for validated targets with prognostic and/or predictive value in screening prostate cancer patients.

The following biomarker categories were the most successfully tested in prostate cancer:

(a) Molecular variants of PSA (e.g., f/t PSA ratio) which are markers of malignancy, able to discriminate prostate cancer from BPH and markers of tumour aggressiveness as well. Two diagnostic tests based on quantification of PSA variants (PHI and 4K) have received FDA approval for discriminating benign conditions from prostate cancer and identifying aggressive tumours.
(b) Quantitative and phenotypical analysis of CTCs and their DNA content as well as cfDNA proved to be indicative for tumour aggressiveness and risk of distant metastasis and according to some studies as therapy-response markers. These markers are particularly important in identifying tumour heterogeneity and the clonality of metastases. The CellSearch™ method is an FDA-approved technology based on CTC characterisation used to predict outcome of prostate cancer patients.
(c) Blood miRNAs either free or within EVs. While a high number of miRNAs are proposed as candidate biomarkers there is an increasing consensus across different studies about the following miRNAs: miR-141, miR-145 and miR-375, which are markers of malignancy (discriminating prostate cancer from BPH), risk prediction, metastasis or relapse indicators. Importantly, recently miRNAs have been correlated with response to radiotherapy and prediction of radiotherapy-related toxicities as well. The application of miRNAs as biomarkers in prostate cancer is still in experimental phase despite the very numerous studies published in this topic. A common characteristic of the studies is that they are mostly local initiatives with low patient numbers (see Table 1). While miRNAs are clearly very promising markers, discrepancies in the findings of the different studies do not allow their validation and consequently their transition into the clinic. It is important to mention that assaying miRNA panels for screening from blood (or urine) is a non/minimally invasive and fast method, which is suitable for high-throughput screening and it is cost efficient. Thus, miRNAs could become ideal biomarkers.
(d) Immune and inflammatory markers. A large panel of soluble molecules, mainly cytokines, chemokines or growth factors were correlated in different studies with response to radiotherapy, prediction of tumour radioresistance and patient radiosensitivity as well as predisposition to radiotherapy-related toxicity. These markers are still in experimental phase despite significant efforts invested in better understanding local and systemic immune responses in prostate cancer. Since immunotherapy is rapidly becoming part of the everyday treatment routine, it is extremely important to find suitable markers able to identify patients responsive to immunotherapy.
(e) Gene expression signatures and gene polymorphisms indicative of disease progression and therapy response analysed either in traditional biopsy material or in CTCs from liquid biopsies. Due to differences in gene expression signatures in prostate cancer between European American men and African American men, care must be taken in the interpretation of these genetic traits in African American men. Gene expression panels under development already take into account racial differences, using markers with similar predictive values between European American and African American men [211].

The major requirements and guidelines for biological parameters to be considered as biomarkers and to be clinically approved have been extensively described elsewhere [212,213]. Basically, the procedure of biomarker development should adhere to the REMARK guidelines. Besides their proven clinical relevance, proposed biomarkers should fulfil strict specificity and sensitivity criteria, also they have to be reproducible, easy-to-perform and to interpret and cost-effective. Despite the high number of promising biomarker candidates in prostate cancer very few have actually been approved for clinical use and their spread in the clinical routine is very slow. We believe that the most important reason for this relatively low transition of experimental biomarkers into clinical setting is that most studies stop at the discovery or qualification phase and fail to proceed to biomarker verification and validation. In Table 1 one can see that many of the listed clinical trials recruited very low patient numbers, which were not sufficient for validation purposes. A large-scale validation study of a prioritized biomarker needs substantial financial and collaborative efforts involving multi-institutional and optimally international collaboration, which might take years to be finalized. Within the European Union EU-supported multi-national collaborative projects could be one solution for wide-scale harmonized biomarker validation studies. Additionally, given the wealth of available data on biomarker candidates, a meta-analysis would help in biomarker prioritisation, highlighting the most promising targets for large-scale validation.

## Data Availability

Not applicable.
